# Plant Proteins: Assessing Their Nutritional Quality and Effects on Health and Physical Function

**DOI:** 10.3390/nu12123704

**Published:** 2020-11-30

**Authors:** Steven R. Hertzler, Jacqueline C. Lieblein-Boff, Mary Weiler, Courtney Allgeier

**Affiliations:** Scientific and Medical Affairs, Abbott Nutrition, 2900 Easton Square Place, Columbus, OH 43219, USA; jacqueline.boff@abbott.com (J.C.L.-B.); mary.weiler@abbott.com (M.W.); courtney.allgeier@abbott.com (C.A.)

**Keywords:** plant protein, protein quality, PDCAAS, DIAAS, vegetable protein, protein requirements, amino acids

## Abstract

Consumer demand for plant protein-based products is high and expected to grow considerably in the next decade. Factors contributing to the rise in popularity of plant proteins include: (1) potential health benefits associated with increased intake of plant-based diets; (2) consumer concerns regarding adverse health effects of consuming diets high in animal protein (e.g., increased saturated fat); (3) increased consumer recognition of the need to improve the environmental sustainability of food production; (4) ethical issues regarding the treatment of animals; and (5) general consumer view of protein as a “positive” nutrient (more is better). While there are health and physical function benefits of diets higher in plant-based protein, the nutritional quality of plant proteins may be inferior in some respects relative to animal proteins. This review highlights the nutritional quality of plant proteins and strategies for wisely using them to meet amino acid requirements. In addition, a summary of studies evaluating the potential benefits of plant proteins for both health and physical function is provided. Finally, potential safety issues associated with increased intake of plant proteins are addressed.

## 1. Introduction

Protein is a nutrient that has been trending increasingly positive in the minds of consumers, with demand rising for both plant and animal sources of protein [1]. In addition, there is a growing body of clinical evidence, especially in older adults, supporting health benefits associated with protein at or above current dietary protein intake recommendations. Among these health benefits are increases in lean body mass [2,3,4,5,6], functional benefits such as increased leg power [4] or gait speed [6], and improved bone density [7,8,9]. Thus, on the one hand, there is likely to be a continued push for protein-rich options in the food marketplace. On the other hand, the global production of an increased volume of food protein, especially high-quality animal protein, could present environmental sustainability challenges. The production of 1 kg of high-quality animal protein requires feeding 6 kg plant protein to livestock, which introduces the subsequent strain on land and water resources, as well as potential increases in greenhouse gas emissions, associated with livestock agriculture [1,10]. Wider and prudent use of plant proteins in the diet can help to supply adequate high-quality protein for the population and may reduce the potential for adverse environmental consequences. This review presents information on: (1) the nutritional quality of plant proteins; (2) strategies for wisely using plant proteins to meet indispensable amino acid requirements; (3) effects of plant proteins on health and physical function; and (4) potential health and safety concerns associated with plant proteins.

## 2. Determination of Protein Quality

Two requirements for a protein to be considered high quality, or complete, for humans are having adequate levels of indispensable amino acids (see Table 1) to support human growth and development and being readily digested and absorbed.

**Figure 1:**
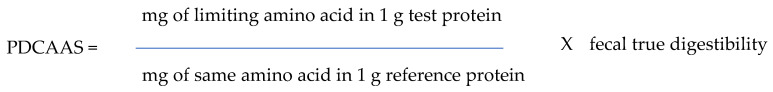
Calculation of the PDCAAS (adapted from [15]).

Various methods for evaluating protein quality have been developed over the years, but amino acid scoring is currently the recommended method by the Food and Agricultural Organization of the United Nations (FAO) and the U.S. National Academy of Sciences [11,12]. The Protein Digestibility Corrected Amino Acid Score (PDCAAS) was developed in 1989 by a Joint FAO/WHO Expert Consultation on Protein Quality Evaluation [13] to compare the indispensable amino acid content of a test protein (mg/g protein) to a theoretical reference protein thought to meet indispensable amino acid requirements (mg/g protein) for a given age group, creating a ratio known as the amino acid or chemical score. The indispensable amino acid with the lowest ratio is referred to as the most limiting amino acid. The most limiting amino acid score is corrected for the fecal true digestibility of the protein. To determine fecal true protein digestibility, rats are fed a known amount of nitrogen from the test protein and then fecal nitrogen excretion is measured [14]. This measure represents apparent protein digestibility. The fecal nitrogen excretion from the rats on a protein-free diet is then subtracted from fecal nitrogen excretion on the test protein, which accounts for non-dietary protein nitrogen excretion from bacterial cells and digestive secretions. The result is referred to as true fecal protein digestibility. The calculation equation for the PDCAAS is shown in Figure 1.

The results can be expressed as either decimals or multiplied by 100 to be expressed as a percent. A PDCAAS of <1.00 indicates that the protein is suboptimal and PDAAS scores >1.00 are truncated to 1.00.

In 2011, the FAO introduced an updated amino acid scoring system, the Digestible Indispensable Amino Acid Score (DIAAS) [16]. The DIAAS is calculated and interpreted similarly to the PDCAAS, but with a few important differences. First, the reference patterns for the indispensable amino acids were revised to reflect advances in the scientific knowledge regarding amino acid requirements. Second, a single estimate of fecal protein digestibility is no longer used. Rather, the concept of the ileal individual amino acid digestibility was incorporated. True fecal digestibility of protein, which is based on nitrogen excretion in the feces, is complicated by the considerable exchange of protein, amino acids, and urea between systemic pools and the lower gastrointestinal tract. In response to this limitation, it was recommended to measure ileal amino acid digestibility, which reflects the concentration of amino acids that reaches the ileum and would hence enter the colon, derived from ileostomy output studies conducted in animals or humans. As such, each indispensable amino acid from a given protein source will have an associated ileal digestibility value and its amino acid score will be corrected for that value. Finally, unlike the PDCAAS, the DIAAS method allows for scores >1.00 to acknowledge that there may be incremental health benefits associated with these higher DIAAS scores.

## 3. The Quality of Plant Proteins

In general, most animal-based protein sources, such as milk, whey, casein, eggs, and beef, have PDCAAS at or very near 1.00 [13,17,18]. As such, they are generally considered complete protein sources for supporting indispensable amino acid requirements for human growth and development. Plant proteins, however, may have insufficient levels of one or more indispensable amino acids. Legumes are frequently low in the sulfur-containing amino acids methionine and cysteine, while lysine is typically limiting in grains [19]. However, it should be noted that plant proteins differ regarding the amounts of limiting amino acids that are present. Table 2 shows the PDCAAS and DIAAS ratings for milk protein, whey, and several selected plant protein sources. Similar to milk protein and whey, soy protein essentially has a PDCAAS of 1.00, and four more proteins (canola, potato, pea, and quinoa) have a PDCAAS of at least 0.75.

While the PDCAAS of most plant proteins may be less than 1.00, the individualized protein scoring system is only one way to evaluate the potential contributions of a protein to the diet. Canada uses a method based on the Protein Efficiency Ratio (PER), which is growth/weight gain assay on rats fed different protein sources. Health Canada provides a list of PER values for different protein foods on their website and suggests that the PER of a protein source can be estimated by multiplying the PDCAAS by 2.5 [58]. Several other factors can increase the potential contribution of plant-based proteins to meeting overall dietary protein and indispensable amino acid needs. One aspect to consider is the amount of dietary protein contributed by a specific plant protein source. In the case of plant versus animal proteins, simply consuming more of the plant protein can help to provide higher indispensable amino acid intakes. Given that many whole food sources of plant-protein are less calorie-dense than animal sources of protein, greater overall food intake is needed to meet energy requirements which, in turn, helps meet indispensable amino acid requirements. In addition, it has now become much easier for consumers to boost intake of plant proteins via the availability of multiple plant-based protein isolates and concentrates (soy, pea, canola, potato, fava, etc.) in the food industry. It was once difficult for individuals to take in relatively large amounts of protein from whole plant foods because they typically have a low percentage of protein. However, plant protein isolates and concentrates, which often contain 80% or more protein by weight, make it possible to consume 10–20 g or more of plant-based protein per one serving of a ready-to-drink shake or powder mix.

Dietary protein variety is also key for meeting indispensable amino acid requirements. While the PDCAAS of an individual protein is critical when evaluating the quality of a sole-source protein, it becomes less significant when the diet contains proteins from many sources. For example, lysine is often limiting in grain proteins, but such proteins are good sources of the sulfur-containing amino acids. On the other hand, legumes are often rich sources of lysine but are limiting in sulfur-containing amino acids. Consumption of these two protein sources over the course of the day allows them to “complement” one another, helping to meet requirements for both types of indispensable amino acids. A classic example would be a combination of pea and rice proteins. Protein blends of pea and rice ranging 40–90% pea protein can achieve a PDCAAS of 1.00, using the 2011 FAO amino acid reference pattern for adults [16]. Flexitarian approaches, in which persons consume increased amounts of plant-based proteins but also include some animal proteins, represent another strategy for helping to meet indispensable amino acid requirements. Thus, the quality of protein in the diet may be quite high if the plan is to consume a variety of plant proteins with differing amino acid profiles.

One question that has arisen for vegetarians is whether it is needed to combine complementary protein sources at the same meal. Young and Pellet [19] addressed this issue. They noted that the common limiting amino acid in grains, lysine, has a significant pool in the skeletal muscle. After a protein-rich meal, they estimated that 60% of the adult daily requirement for lysine could be stored in this pool within 3 h. If a person were to consume a lysine-poor meal within 3 h of a lysine-rich meal, there would still be adequate intracellular lysine available to promote protein synthesis. Thus, it is not necessary to consume complementary protein sources at the same meal if the gap between meals is relatively short, around 3 h; the complementary amino acids will be metabolically available for protein synthesis.

**Figure 2:**
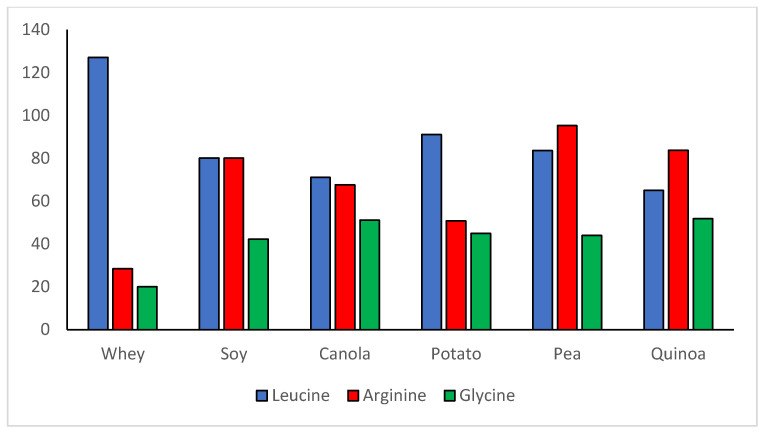
Comparisons of leucine and selected dispensable amino acid concentrations (mg/g protein): whey versus the Top 5 highest quality plant proteins in Table 2.

An often-neglected aspect of plant proteins is their high content of some important dispensable/conditionally indispensable amino acids. The PDCAAS method of evaluating protein quality focuses only on indispensable amino acids and generally on whole body protein requirements. However, since the development of the PDCAAS concept, the knowledge base around the health- or performance-related effects of individual amino acids, both indispensable and conditionally indispensable has grown dramatically. For example, whey protein has received much attention for muscle building due to its high level of leucine (see Figure 1), which serves as a nutrient signal for initiating the process of muscle protein synthesis [59,60]. However, it is important not to forget the vital physiologic functions of dispensable/conditionally indispensable amino acids found in large amounts in plant proteins. Soy protein, while not as high as whey in leucine, is nearly three times higher in arginine, 2–3 times higher in glutamine, and has double the glycine content (Figure 2 and Table 3). Other plant proteins can be high in these amino acids as well. Arginine is necessary for the body’s synthesis of nitric oxide (vasodilator) and creatine, for urea cycle function, for regulating hormone secretion, and for immune function [61,62]. Glutamine is a primary fuel source for rapidly proliferating cells such as those in the immune system and gastrointestinal tract and functions in the synthesis of arginine, ornithine, and several other compounds [61,63]. Glycine is critical for collagen synthesis, comprising up to 1/3 of the amino acids in collagen and some studies suggest that its biosynthesis in humans may not be adequate to meet requirements [64,65,66,67]. Although amino acids such as arginine, glutamine, and glycine might not be classified all the time as indispensable amino acids, they perform many critical functions and plant proteins can be significant sources. Thus, the content of these dispensable/conditionally indispensable amino acids deserves to be taken into consideration when evaluating the value of plant proteins in the diet.

## 4. Importance of Plant Proteins in Health

The benefits of plant proteins on long-term health and chronic diseases have been a trending topic in recent years. This section summarizes some of the most recent evidence and analytical reviews for several target health areas, including cardiovascular health, metabolic syndrome, diabetes, cancer, renal function, lean body mass, and strength, as well as overall morbidity and mortality. This section is not meant to be a comprehensive review of the health effects of plant protein. Rather it is meant to highlight key recent studies and meta-analyses and open a dialogue to suggest future areas for research. 

### 4.1. Plant Protein and Cardiovascular Disease and Metabolic Risk Factors

Numerous studies have explored the potential impact that dietary plant proteins have on reducing cardio-metabolic risk factors. One of the first reports to synthesize the results of plant protein intake as a substitution for animal protein was a study published in 2017. In this systematic review and meta-analysis of 112 randomized clinical trials across adults with and without hyperlipidemia, the authors demonstrated reduced markers of cardiovascular disease in favor of plant protein over animal protein consumption [72]. The authors reported a reduction in blood lipids across the studies, including lower low-density lipoprotein cholesterol, non-high-density lipoprotein cholesterol, and apolipoprotein B. While the authors called for higher quality randomized trials to confirm their results, this evidence supports plant protein as an effective substitution for animal protein in the diet to help reduce cardiovascular disease risk factors in adults. A more recent meta-analysis was performed on the impact of plant protein compared with animal protein across 32 intervention trials in hypercholesterolemic patients [73]. While there was evidence in favor of plant proteins to lower lipid profiles, most trials in this analysis examined soy products as the intervention compared with a variety of animal protein sources. Therefore, it may be difficult to draw a broad conclusion about all plant proteins based on the limited types of plant proteins studies and on potential confounding effects driven by other bioactive properties of soy products. 

Benefits of plant proteins and metabolic health have also been described for adolescent populations. Obesity is a growing problem worldwide among adolescents, and several studies have examined the potential benefits of plant protein intake in relation to obesity, weight management, or metabolic syndrome. One such study was the Healthy Lifestyle in Europe by Nutrition in Adolescence (HELENA) study, a cross-sectional study of European adolescents [74]. In this study, both total and animal protein intake were higher in obese adolescents. Adolescents consuming higher levels of plant protein exhibited lower body fat percentages and BMI compared with those adolescents with higher animal protein intake. However, protein is critical for many physiologic functions and facets of development, and adequate protein intake is important. The study suggested increasing plant protein in adolescent diets as a substitution for animal protein to help control obesity and for its potential positive benefits for cardio-metabolic factors [74]. Incorporating more plant proteins into the diet to take the place of excess calories and animal protein may be a useful strategy to assist with adolescent obesity.

Criticism has arisen from some researchers, however, regarding the attempt to make blanket statements about the superiority of the cardio-metabolic health benefits of plant proteins versus animal proteins. While advantages of plant food sources have been described, researchers advise not to indiscriminately consider all animal proteins as inferior to plant protein for cardiovascular health, citing limited and inconsistent evidence to support that type of conclusion [75,76]. In an editorial, Campbell cautioned that not all studies have shown a detrimental effect of red meat compared with plant protein on cardiovascular disease risk markers and suggested there is mixed evidence when evaluating white meat as compared to red meat as a healthier animal option [75]. For example, the randomized, crossover, controlled trial by Bergeron et al. [77] found a benefit of non-meat protein intake over animal protein intake, but no difference between white meat versus red meat in the animal protein dietary periods. The study authors concluded that more plant-based protein should be introduced into the diet to reduce cardiovascular disease risk but noted that their short intervention period and inability to show a difference between various animal protein diets may limit the interpretation of results. In summary, lumping all animal proteins together as being inferior to plant proteins regarding cardiovascular disease risk is not advised. Generalizing the health benefits of plant protein over animal protein is difficult due to trial inconsistencies and limited control of variables. The overall health composition of foods should be considered instead of creating competition between animal plant-based protein sources, and a wide variety of nutritious protein-rich foods from animal and plant sources should be incorporated into the diet along with healthy dietary habits [75].

### 4.2. Plant Protein and Diabetes

While vegetarian diets are associated with a substantial risk reduction for diabetes [78], it is unclear if substitution of plant protein for animal protein helps to drive this risk reduction. Malik et al. [79], analyzing data from the Nurses’ Health Study II, found that substituting 5% of energy intake from vegetable protein for animal protein was associated with a 23% reduced risk of type 2 diabetes. An acute feeding of 20 g yellow pea protein, served in a tomato soup 30 min in advance of an ad libitum pizza meal, reduced the glycemic response to the pizza meal and the energy intake from the pizza meal (when compared with tomato soup not containing pea protein) [80]. In a similar study, a 400-kcal breakfast comprising a meal replacement beverage containing about 29 g soy protein was compared with an isocaloric, higher glycemic index, lower protein breakfast. The soy protein beverage was associated with: (1) lower postprandial glycemic and ghrelin responses to the breakfast; and (2) decreased postprandial insulin secretion from a standardized lunch fed 4 h later [81].

In a 2015 meta-analysis of randomized control trials that replaced animal protein sources with plant protein for at least 35% of total dietary protein intake over a median study length of eight weeks, the authors reported significant, but modest, improvements in HbA1c, fasting glucose, and fasting insulin levels in individuals with diabetes [82]. These results were positive, but limitations were noted. The authors called for longer and larger clinical trials to confirm results as sample sizes were relatively small in the studies reviewed. It should also be noted that the meta-analysis included reported studies for both type 1 and type 2 diabetic populations. 

Since this meta-analysis was conducted in 2015, a prospective clinical trial evaluated the potential benefits of high protein diets using either plant or animal protein sources in adult individuals (aged 64.3 ± 1 years) with type 2 diabetes. This randomized clinical study of 37 diabetic individuals placed on either a high animal protein diet (80.2% of total protein in intervention) or a high plant protein diet (72.3% of total protein in intervention) for six weeks found that both diets similarly reduced body weight, BMI, HbA1c, and blood lipid markers after the intervention [83]. The animal protein group experienced a decrease in fasting glucose and whole-body insulin sensitivity from baseline, but there was no difference between the protein groups. Further analyses of results from this trial revealed similar responses from both intervention groups for liver fat, markers of hepatic inflammation, and insulin resistance [84]; oxidative stress biomarkers [85]; and pro-inflammatory cytokines [83]. Other studies have also failed to show a benefit of a vegetarian diet over animal protein diets for individuals with diabetes. In a randomized controlled trial in patients with type 2 diabetes, no benefits or differences were observed in cardio-metabolic biomarkers across three groups randomized to a red meat protein diet, soy legume protein diet, or a non-soy legume protein diet after eight weeks [86]. The authors concluded that impact of whole diet could be more relevant than the impact of protein source, and that animal protein could be consumed as part of a balanced diet. Larger and longer-term studies in individuals with diabetes are warranted.

### 4.3. Plant Protein Intake and Incidence of Cancer

Another area of interest for examining benefits of increasing plant protein intake in place of animal protein is in cancer risk reduction. Certainly, the risk for developing cancer is influenced by multiple factors, such as genetic predisposition, environment, and dietary and other lifestyle habits. One group has focused on examining the risk of colorectal cancer in individuals using gene–environment interaction analyses, incorporating several lifestyle factors, genetic factors, and cancer risk [87]. In their examination of a large, prospective Danish cohort, the authors reported an association between certain genetic polymorphisms for fatty acid metabolism and colorectal cancer, which were further associated with high meat intake. They described that high meat intake was associated with high risk of colorectal cancer among some gene carriers compared with those having the same genetic polymorphism who consumed diets with lower meat intake [87]. Therefore, substituting plant protein for animal protein in the diet may be a strategy to lower the risk of colorectal cancer in individuals with certain gene variants. There have been mixed results, however, regarding whether shifting from animal protein to more plant protein will reduce colorectal cancer risk. For example, a recent study included 79 pre-diabetic adults on a one-year weight-loss dietary intervention [88]. This study examined total dietary protein intake, red meat intake, and animal to plant protein ratio. At baseline and after the one-year intervention, these dietary habits were compared with the level of fecal ammonia concentrations, a biomarker for colorectal cancer risk. While this study did report a dose-dependent association between fecal ammonia concentration and the amount of red meat intake, there was no associations between fecal ammonia and total protein intake or the ratio of animal to plant protein in these individuals [88]. 

In our review of the literature, there was limited evidence to confirm the benefits of plant protein above animal protein on its impact on cancer risk reduction. This will likely be a growing area of focus for future research to better understand if plant protein itself confers any benefits or whether the adoption of better dietary habits associated with increased plant protein intake helps to drive favorable health outcomes.

### 4.4. Plant Proteins as Functional Foods

Plant proteins have also been studied for their potential as functional foods. Numerous studies have been conducted to examine the impact on cardiovascular risk, glycemia, or satiety. Many studies have focused on the functional and bioactive properties of soy protein, especially for reducing cardiovascular disease risk, modulating inflammation, or modulating the immune system [89]. A recent systematic review examined the bioactive properties of plant protein sources other than soy, including protein from pea, lupin, fava bean, rice, oat, hemp, and lentil [90]. Most trials reported the benefits of plant protein ingredients by examining postprandial concentrations of blood glucose, insulin, and/or appetite regulating hormones. While there was heterogeneity in results, studies that compared animal to plant protein showed no benefit of plant protein on regulating postprandial glycemia. Similarly, the benefits of plant protein as a functional food for satiety showed mixed results, although there may be some benefit to pea protein. It is likely that the bioactive components of a plant diet are often attributed to whole food sources than isolated protein. It is well known that numerous components in plants, such as carotenoids and flavonoids, confer bioactive benefits for health. However, further research on plant proteins and bioactive peptides is needed.

### 4.5. Plant Protein Intake and Its Relationship to Mortality

Many studies have also linked sources of protein intake to mortality. A recent publication from the large, prospective cohort from the NIH-AARP Diet and Health Study also examined the impact of dietary protein choice on mortality [91]. In this study, 617,199 individuals aged 50–71 across the U.S. were followed from 1995 or 1996 until study follow-up in December 2011. Intake of plant protein was significantly inversely associated with all-cause mortality as well as cause-specific mortality from cardiovascular disease and stroke in both males and females. They reported that replacement of just 3% of protein intake with plant protein versus animal was associated with a 10% reduction in overall mortality across both men and women [91]. These results are consistent with a recent systematic review and meta-analysis on the impact of protein intake on mortality risk [92]. Aligned with other reports highlighting the importance of increased protein intake, especially as we age, higher total protein intake was associated with a reduced all-cause mortality risk. Stratifying data into animal protein intake versus plant protein intake, however, revealed a lower all-cause mortality risk for those consuming plant protein diets. Ten studies examining animal or plant protein intake were analyzed in the meta-analysis for the association with mortality from cardiovascular disease. While there was no clear association between animal protein intake and mortality, an inverse association was found between plant protein intake and cardiovascular disease risk. These studies support a benefit of substituting more plant protein into the diet in place of animal protein in terms of longevity and mortality.

### 4.6. Renoprotective Effect of Plant Proteins

The American diet is typically characterized as low in fruits, vegetables, dairy, and healthy oils and exceeds recommendations for total grains, total protein foods, added sugar, saturated fats, and sodium [93]. This diet, also characterized as the Western diet, has been under scrutiny to establish the metabolic differences that contribute to chronic disease, especially regarding chronic kidney disease (CKD) [94]. Recent epidemiological evidence suggests that not only the amount of protein, but also the origin of protein (e.g., plant vs. animal), may be a factor that influences kidney function [95]. The nuances of earlier experimentation with low versus normal recommended protein intake lent clues to the potential impact of protein origin. Viberti et al. [96] replaced the animal protein in an isocaloric diet with vegetable sources in a crossover study with healthy adults and observed a reduction in glomerular filtration rate (GFR) and renal plasma flow (RPF). A sub-study within a broader investigation that was designed to examine the effect of dietary protein on GFR compared healthy vegetarian subjects with those on an omnivorous diet. Both groups ate their normal diets ab libitum. The mean plasma creatinine level was not significantly different between groups, but the creatinine clearance was significantly lower in the vegetarian group [97]. A soy protein-rich diet was found to reduce glomerular hyperfiltration in a study of patients with type 1 diabetes with early stage nephropathy [98]. Increases in GFR and glomerular hyperfiltration contribute to the incidence of kidney injury and indicate how diet can have a negative impact on kidney function [99]. 

The effect of plant and animal protein intake on renal function continues to be explored. In a prospective analysis of a large cohort (*n* = 15,055) from the Atherosclerosis Risk in Communities (ARIC) study [100], dietary renal acid load was positively associated with chronic kidney disease (CKD) incidence (defined by the authors as 25% reduction of estimated glomerular filtration rate (eGFR), CKD related hospitalization, end-stage renal disease, or mortality). This mirrors the findings of a 10-year longitudinal cohort study where the objective was to assess the source of protein intake in a cohort of older women and possible link to incidence for age-related rate of renal function decline. Greater consumption of plant protein was related to slower declines in eGFR, but intake of animal protein was not associated with kidney function decline [95]. In two one-year intervention studies, patients with stage 3 or 4 CKD were treated with either sodium bicarbonate or fruits and vegetables dosed to reduce renal acid load, a hypothetical metabolic risk factor for kidney damage, by 50% [101,102]. Both treatments ameliorated metabolic acidosis and indices of kidney injury and did so without producing hyperkalemia. In another trial, participants on diets with equivalent nutrient content had lower serum phosphorus and phosphorus excretion when the protein source was vegetarian as compared to animal-based [103]. The observational outcomes of the Chronic Renal Insufficiency Cohort Study supported these findings to indicate an association between plant protein consumption and reduction in metabolic risk factors for CKD exists [104]. The totality of this evidence points to the benefit of plant-proteins in the diet to lessen the impact of protein intake in patients with increased protein needs, due to wasting, from glomerular hyperfiltration. 

The plant-based proteins from soybean and rice endosperm have demonstrated renal protective properties in diabetic rat models [105]. One potential mechanism of action for the renoprotective effect of plant protein is an indirect effect mediated by improved glucose homeostasis, with plant protein intake being associated with reduced fatty liver development. Another potential explanation is that a protein such as rice endosperm protein is high in arginine, a precursor of nitric oxide (NO), which is depleted in this rat model [105,106]. The improvement of renal hemodynamics which results from the supplementation of arginine could be the direct result of an increase in NO production [106]. These plant protein sources bring additional compounds into the mix that need to be considered as well, such as soy isoflavones, which might affect renal function through cell signaling actions and nitric oxide production affecting renal perfusion [107]. Soy consumption has also been associated with improvements in antioxidant status and systemic inflammation in CKD patients [107]. Put another way, the whole “protein package” should be considered in terms of health benefits. Soy’s effects on renal function could be the result of the whole food’s impact on risk factors for CKD such as dyslipidemia, hypertension, and hyperglycemia [108]. In summary, other factors such as fiber and phytochemicals may play a role in renal protection in whole food plant-based diets; however, these components cannot be fully responsible for the renal benefits seen in the studies using protein isolates. In diets high in whole plant foods, it is more likely that the positive effect on renal function is due to synergistic effects from plant-protein and from other plant components. This renoprotective effect is the basis for recommending the incorporation of high-quality plant proteins not only in the diet of those with renal insufficiency [109], but also the general population.

### 4.7. Plant Proteins for Lean Body Mass and Strength

Meeting total daily protein needs is important for persons engaging in either strength or endurance training. In addition, the concept of reaching meal total protein and leucine content thresholds of 20–40 and 2–4 g, respectively, several times per day to promote “maximal” muscle protein synthesis (MPS) [60,110,111,112,113,114] has become popular among active persons (young and old). Most studies examining the effects of meal protein dose on muscle protein synthesis, especially post-resistance training, fed high-quality animal proteins such as dairy (e.g., whey and casein) or egg protein. Tang et al. [115] studied the effect of feeding whey hydrolysate, soy protein, and casein, matched to provide 10 g indispensable amino acids, on mixed MPS at rest and over a 3-h period following unilateral leg resistance training. Postexercise, the whey hydrolysate promoted significantly greater MPS than did either soy or casein. However, the soy protein outperformed casein at rest and postexercise. Further, even though it was significantly lower, the postexercise MPS fractional synthetic rate (%/h) for soy protein was still about 80% that of whey. The authors attributed this finding to either differences in the rates of digestion of the three proteins or their leucine content. Because soy protein has a lower percentage of leucine (~8%) compared with whey protein (~12%), it is possible that simply providing a little bit more soy protein to reach the critical leucine threshold is all that is needed to promote comparable levels of postexercise MPS between the two proteins. 

Studies with other plant proteins tend to bear this out. In a sample of young women also performing unilateral leg resistance training, increasing protein intake to double the RDA from potato protein elevated both resting and exercise-associated 24-h MPS above the baseline level [28]. Curiously, in this study, supplementation with an isocaloric carbohydrate placebo also caused comparable increases in MPS in both the resting and postexercise state, so the true benefits of the potato protein were unclear. In another study, ingestion of 35 g micellar casein by older men (non-exercising) versus 35 g wheat protein hydrolysate caused greater increases in MPS in the 4-h postingestion period [116]. However, upping the dose of wheat protein hydrolysate to 60 g resulted in rates of MPS that exceeded that of 35 g whey protein and were comparable to that of 35 g micellar casein.

Acute measures of MPS occurring a few hours after the ingestion of protein have questionable value in predicting longer-term gains in lean body mass with training [117]. Gaining muscle mass is a complex process affected by a variety of physiological factors, so actual training studies evaluating the influence of protein supplementation on muscle mass and strength gains over time are needed to better assess the value of plant proteins for muscle building. Some resistance training studies (12–36 weeks) in young adults have reported that fluid milk or whey protein is superior to soy milk or soy protein for muscle mass and strength [118,119]. However, a recent meta-analysis of nine resistance-training studies with a total of 266 participants [120] was conducted to evaluate the effects of matched protein doses from soy versus animal proteins on muscle mass and strength outcomes. Of the nine studies in the meta-analysis, five compared whey with soy, while four compared soy with other proteins (beef, milk, or dairy protein). Subjects included both young (18–38 years) and older (61–67 years) adults and the duration of training ranged 6–36 weeks (2–5 times per week). Amounts of protein supplemented to the diet ranged 18–85 g/day. There were no differences between soy protein and the animal proteins for improvements in bench press strength, squat/leg press strength, or lean body mass outcomes. 

Training studies have also reported positive outcomes for other plant proteins than soy. Joy et al. [121] reported that 48 g/day of rice or whey protein isolate on training days during an eight-week resistance training program in college-aged adults caused similar improvements in body composition and bench and leg press strength. A study in elite mixed martial artists undergoing six weeks of intense training demonstrated no differences between 75 g/day of whey or rice protein isolate on body composition outcomes [122]. In addition, pea protein supplementation (25 g twice/day) was shown during 12 weeks of resistance training to increase biceps muscle thickness to the same degree as an equivalent amount of whey protein [123]. Likewise, Banaszek et al. [124] supplemented participants in a high-intensity functional training program over eight weeks with 48 g/day of either whey or pea protein and observed that both proteins resulted in similar body composition, muscle thickness, force production, workout performance, and strength. Finally, a meta-analysis of the effects of protein intake on resistance training outcomes concluded that the major considerations for protein intake were to achieve an intake of 1.6 g/kg body weight per day, separating it into 0.25 g/kg doses [125]. Of minor importance were factors such as timing of intake, postexercise protein dose, and protein source. Part of the explanation for differences in efficacy between plant and animal proteins may have to do with whether short-term (e.g., MPS) compared with long-term (e.g., increases in actual lean body mass) outcomes.

Whey protein is quite effective for promoting increases in both short-term measures of MPS and resistance-training induced gains in lean body mass and strength and, largely due to its high leucine content, can lead to these improvements in lower doses (<30 g/day) [119] than might be achieved with plant proteins. However, supplementing with larger doses of plant proteins (40 g/day or higher) can provide similar fitness outcomes to those achieved with whey protein. The wider availability of plant-based protein concentrates and isolates now makes it easier to achieve these higher plant protein intakes for those who wish to push the balance of their protein intake more heavily toward plant-based sources.

Another point of importance is the value that might be achieved by combining plant and animal proteins in a supplementation program to take advantage of the relative strengths of each kind of protein. For example, PER determinations in rats with 30:70 animal:plant protein ratios have shown that, for several animal and plant protein combinations, the 30:70 ratio resulted in equivalent or greater PER scores than did the animal protein at 100% [126]. Similarly, two studies of a protein blend (20 g) containing 25% whey protein isolate, 25% soy protein isolate, and 50% sodium caseinate can promote MPS to a level equivalent to whey protein alone and may be associated with more prolonged positive amino acid net balance (i.e., arteriovenous differences in the leg) compared with whey protein [127,128]. Thus, for those individuals who want to incorporate plant proteins but are still open to animal proteins as well, it is possible to put them together to achieve the desired results.

## 5. Health Concerns Associated with Plant Proteins

### 5.1. Antinutrients

One health concern associated with increased dietary intake of plant-based proteins is the presence of antinutrients in plant foods. Antinutrients are natural substances produced by plants that can interfere with the digestion, absorption or utilization of nutrients in food and may have other adverse effects as well [129]. Antinutrient adverse effects may include leaky gut and autoimmune effects (e.g., lectins and some saponins), protein maldigestion (trypsin and protease inhibitors), carbohydrate maldigestion (alpha-amylase inhibitors), mineral malabsorption (phytates, tannins, and oxalates), interference with thyroid iodine uptake (goitrogens), gut dysfunction, inflammation, and behavioral effects (conversion of cereal gliadins to exorphins) [129]. Often, the adverse effects of antinutrients have been observed in animals fed unprocessed plant proteins and these observations have triggered fears in people regarding the consumption of some plant foods. However, it is important to note that antinutrients are not always associated with adverse effects and, in some cases, their effects on the body may be positive. At low levels, phytates, lectins, phenolic compounds, enzyme inhibitors, and saponins may help to reduce blood glucose and/or plasma cholesterol and triglycerides [129]. Saponins may help liver function and reduce platelet agglutination and some saponins, as well as phytates, protease inhibitors, lignans, and phytoestrogens, may reduce cancer risk [129]. In addition, tannins may have antimicrobial effects [129]. As such, some of the health benefits of plant-based diets may be attributed to the presence of low levels of these “antinutrients”. Finally, multiple pathways exist for greatly reducing the concentration of antinutrients in plant proteins, including soaking, fermentation, sprouting (germination), heating, gamma irradiation, and genomic technologies [129]. Food processing techniques make it possible to largely remove antinutrients such as glucosinolates, phytates, erucic acid, and insoluble fiber from canola/rapeseed proteins, which dramatically improves their bioavailability [26]. Because plant protein concentrates and isolates typically undergo processing to mostly eliminate antinutrients, their digestibility is typically much higher than when the protein remains in the whole food matrix. For example, the protein digestibility of soy protein isolate is 96% or higher, while the protein digestibility of soy flour is only 84% [24,25].

### 5.2. Soy Protein and Isoflavones

Soy protein has been the target of both health promotion claims and potential adverse health effect concerns for some time due to its content of isoflavones. Isoflavones are compounds that have elements of their chemical structure similar to estrogen and some weakly bind with estrogen receptors [130]. The concern has been raised that soy isoflavones might have endocrine disrupting impacts on reproductive hormones, largely based on in vitro cell culture or rodent studies involving large doses of isoflavones [131,132,133]. The isoflavone content of various soy protein ingredients has been reported as follows (wet basis, expressed as aglycones): defatted and whole soy flours (120–340 mg/100 g), soy protein isolates (88–164 mg/100 g), commercial textured soy proteins (66–183 mg/100 g), and soy hypocotyl flours (542–851 mg/100 g) [134]. As a result, consumers may choose to avoid soy protein for fear of adverse effects on reproductive or thyroid hormones. However, multiple lines of research over the last 15 years have shown that concerns regarding adverse hormonal effects from physiological amounts of soy foods in the diet are largely unfounded. In 2015, the European Food Safety Authority conducted a comprehensive evaluation of the safety of isoflavone supplements for peri- and postmenopausal women. The evaluation showed that daily doses of 35–150 mg of isoflavones in this population resulted in no increase in breast cancer risk, no effects on endometrial thickness or histopathological changes in the uterus over 30 months (some nonmalignant histopathological changes at 60 months), and no changes in thyroid hormone status [135]. 

A meta-analysis of 15 placebo-controlled studies of men of varying ages have reported that soy protein intake up to 60 g/day has not been associated with significant alterations in testosterone, sex hormone-binding globulin, free testosterone, or free androgen index [136]. Similarly, Dillingham et al. [137] observed that the feeding of approximately 32 g protein/day for 57 days from either low or high isoflavone soy protein was associated with only minor changes in serum reproductive hormones in young healthy men. In another comparison of low versus high isoflavone soy protein supplementation, the protein supplementation, regardless of isoflavone content, did not influence semen quality parameters (semen volume, sperm concentration, sperm count, sperm mobility, sperm percent motility, total motile sperm count, or sperm morphology) in healthy young men [138].

Because some types of breast cancer may be estrogen-sensitive, the safety of soy for breast cancer patients has been questioned. The issue of whether soy protein/soy isoflavones affects the risk of breast cancer or its recurrence has also been addressed in multiple investigations and reviews. Messina [130] concluded that soy foods do not increase the risk of breast cancer and will not worsen cancer outcomes in women with breast cancer. A meta-analysis in 2016 and two more in 2019 reported similar conclusions and further suggested that soy food intake may be associated with a decrease in the risk of breast cancer and improved breast cancer survival [139,140,141]. A systematic review of 13 prospective cohort studies for primary breast cancer incidence and five prospective cohort studies examining risk of recurrence and mortality (4–7 years follow-up post first diagnosis) [142,143] stated that soy foods do not affect the risk of primary breast cancer, but, in patients with breast cancer, a diet high in soy is associated with a 25% decrease in cancer recurrence and a 15% decrease in mortality. The protective effect of soy was significant in both estrogen receptor-positive and -negative breast cancer types, but the reduction in recurrence was stronger in the estrogen receptor-negative (HR = 0.64; 95% CI 0.44–0.94) compared with estrogen receptor-positive (HR = 0.81; 95% CI 0.63–1.04) breast cancer type. The American Cancer Society supports the intake of soy foods in breast cancer survivors [144].

Potential concerns regarding the effects of soy foods on thyroid function may serve as a barrier to increased soy protein intake among consumers. These questions arose based on some cases of goiter in infants on soy infant formula 60 years ago [145,146] and on in vivo [147] and in vitro [148] research suggesting that isoflavones inhibit the activity of thyroid peroxidase, a key enzyme that, with iodine, helps the thyroid synthesize the hormones triiodothyronine (T3) and thyroxin (T4).

Despite early concerns regarding potential harmful effects of soy on thyroid function, the weight of current evidence points more strongly to the safety of soy. Recently, Otun et al. [149] conducted a systematic review and meta-analysis of 18 studies of the effects of soy foods/isoflavones on thyroid hormone function in adults. There were no overall effects of soy or isoflavones on thyroid function, although the authors did note a modest increase in TSH in some studies that was of unclear clinical relevance. Finally, the absence of an epidemiological association between soy food intake and thyroid function in countries where soy intake is high further argues for the safety of soy. While the possibility of adverse effects of soy on thyroid function cannot be ruled out in some sub-populations (e.g., those with marginal iodine status or sub-clinical hypothyroidism), individuals with normal thyroid function and iodine intake should be able to safely consume soy foods/protein [150].

With regard to hypothyroid individuals on thyroid replacement medication, there is limited case study evidence that soy foods may interfere to some degree with the absorption of levothyroxine in some hypothyroid individuals [151]. However, even in this situation, the reasonable intake of soy foods may still be acceptable if the dose of levothyroxine is either increased or timed such that it does not coincide with the soy intake [150,151].

### 5.3. Plant-Based Protein and Allergenicity

As mentioned above, the trend towards an increase in plant protein consumption stems from available evidence indicating that the source of protein (or, the protein “package”), not just the amount of protein, influences our health. Healthcare professionals are recommending adding different protein sources like soy, beans, nuts, or other plant-based proteins in place of red meat and processed meats to lower the risk of several diseases [152]. As the health food industry has grown, a focus for food manufacturers is the trend towards incorporating more plant-based foods to appeal to consumers. This is a trend not only in adults but also in the pediatric population. Increasingly, parents and caretakers are feeding infants and young children plant-based “milk” alternatives to cow milk [153,154,155,156,157] as well as providing more vegetarian options such as plant-based nuggets and burgers into their children’s daily meal plan. Such dietary choices may have unintended outcomes.

One of these outcomes is allergenicity. A food allergy is an adverse health effect resulting from a specific immune response that occurs reproducibly on exposure to a given food [158]. The health effect, called an allergic reaction, occurs because the immune system attacks food proteins that are normally harmless. Symptoms range from mild and transient to severe and life-threatening. According to Food Allergy Research and Education, 32 million Americans are living with potentially life-threatening food allergies. Based on a review of the literature, food allergy is estimated to affect more than 1–2% and less than 10% of the population [159].

In the U.S., more than 170 foods have been identified as triggers of food allergy [158]. The most common foods causing most of the significant allergic reactions include peanuts, tree nuts, fish, shellfish, milk, egg, wheat, and soy [158]. The most common food allergies in children and adults in the United States are allergies to peanut, milk, shellfish, and tree nut, with milk being most prevalent in children and shellfish most prevalent in adults [160,161]. Common food allergens from other countries include: sesame seeds in Canada, European Union (EU), Australia/New Zealand; mustard in EU and Canada; Buckwheat in Japan and Korea; and lupines in the EU [162]. The above have become common food allergens since they are frequently consumed, consumed in relatively large amounts, and consumed in early life stages. As plant protein consumption increases, so will the percentage of allergenic responses for these very reasons. 

Take, for example, lupines. The *Lupinus* genus is closely related to other legumes, such as peanuts, soy, chickpeas, peas, lentils, and beans [163]. In the EU, lupine flour and other lupine protein ingredients were introduced in the 1990s as replacements for soy and wheat [164]. Since its introduction, allergic cross-reactions were noted in some peanut-allergic individuals. This was also observed in Australia, and now lupine is listed on the priority allergen lists by the International Union of Immunological Societies Allergen Nomenclature Subcommittee in the EU and Australia [164].

This is a very similar story to soy protein. Soy originated in southeast Asia and was first domesticated in China around 1100 BC, not being introduced in the U.S. until the 1760s [165]. Tofu and soy sauce were some of the first soy foods for humans. In 1930, soy infant formula was developed, but it was not widely used until the 1950s. In 1959, soy protein isolates were first introduced. From the 1950s, when some milk allergic infants transitioning to soy formula subsequently developed soy allergy, to the 1960s when higher intakes of soy protein in multiple different food sources became possible, the prevalence of soy allergies increased. Even so, soy has demonstrated value as a quality source of plant-based protein. Studies in children have demonstrated that soy supports normal growth and development [166] and improves growth when substituted for other legumes in malnourished children [167,168]. Overall, a wealth of evidence exists to demonstrate soy’s value as part of a healthy and varied diet [169]. All food proteins have the potential to cause allergic reactions, and children tend to be more sensitive to dietary proteins than adults [170]. While soy is a potential allergen in children, soy allergy in children is far less common than allergies to dairy [171], and soy allergy has a prevalence of only 0.4% among American children [172,173] and 0.32% in Canadian children [174]. This compares with prevalence rates of 2.0–3.0% for milk allergy [173,175], 2.0% for peanuts [173], 0.8–2.0% for eggs [176,177], and 1.0% for tree nuts [173,178,179]. Children also tend to outgrow soy allergies over time. One study reported that ~70% of infants with a soy allergy outgrew the allergy by the age of two years [180], and evidence suggests that, by the age of 10 years, only about 1 in 1000 children continue to have a soy allergy [178].

The chemical analysis of plant proteins has been happening for centuries, with the isolation of gluten proteins from wheat dating back over 250 years ago [181]. More recently, increasing emphasis has been placed on the role of plant proteins as allergens, particularly in Europe and the U.S.A., and in relation to novel and transgenic foods [181]. Plant-based food allergens fall into four main families: the prolamin superfamily, cupin superfamily, Bet v 1 family, and profilins. Over 50% of the plant protein allergens fall into two categories, the prolamin and cupin superfamilies [181]. The prolamin family is characterized based on the presence of a conserved eight cysteine amino acid residue pattern CXnCXnCCXnCXCXnCXnC. This stabilizes the protein structure which contributes to overall allergenicity of proteins in this class (highly resistant to heating, proteolysis, and digestion). The major allergens include cereal prolamins, 2S albumins, non-specific lipid transfer proteins, and α-amylase and trypsin inhibitor protein families [182,183,184,185,186].

The prolamin family are seed proteins which include but are not limited to wheat, barley, rye, soybean, rice, maize, and sunflower. Consequently, the prolamin superfamily currently forms the largest and most widely distributed group of plant food allergens [181]. One can visit the Food Allergy Research Resource Program (FARRP) database (http://www.allergenonline.com/) to learn about more different types of allergens. FARRP database contains a comprehensive list of 2171 protein (amino acid) sequence entries that are categorized into 873 taxonomic-protein groups of unique proven or putative allergens (food, airway, venom/salivary, and contact) from 423 species [187].

All protein sources have the potential to have an allergenic effect. As novel plant-based sources of protein emerge into the market, they will inevitably elicit an allergenic response in someone. An example of this is pea protein. Peas are part of the legume family which also includes peanuts, beans, lentils, and soybeans. Due to other plant proteins, such as soy and wheat, having documented allergenic responses, pea protein has been viewed as a potentially less allergenic alternative. Pea protein use as a human food commodity has been steadily increasing in the U.S. Pea protein’s availability, physical and processing characteristics, nutritional value, and low cost have increased its use as a novel and effective alternative to substitute for soybean or animal proteins in functional foods [188]. It can be found in protein powders, medical formulas, and a variety of food substances such as milk, yogurt, cheese, and baked goods. While not common, there have been case studies documented of those with a proven peanut allergy having a reaction to pea protein [189,190]. What is interesting is that plain cooked yellow peas (e.g., entry of peas, split, mature seeds, cooked, boiled, and without salt) average approximately 8% protein by weight [37]. In comparison, current products include pea protein isolates (70–95% protein), concentrates (60–70% protein), and hydrolysates (90–95% protein) [189]. The products listed above provide much higher protein loads than someone eating a serving of cooked peas. It is not surprising, then, that someone not allergic to a serving of whole peas might experience an allergenic response to the much larger doses of pea protein found in products containing pea concentrates and isolates. Although some believe that pea and soy protein have a similar allergenic prevalence [191], pea protein allergenicity has not been extensively studied. While pea proteins are not required to be identified as a potential allergen on food labels in the U.S. or Canada, some have taken notice of pea as a “hidden allergen” [192].

While all dietary proteins are foreign proteins to the human immune system, only a few proteins from plant and animal origin cause an IgE-mediated immune response, typically in a small number of people [162]. Plant protein categories include legumes, nuts and seeds, whole grains, and other (mainly fruits and vegetables). At the time this article was written, there are insufficient data on all plant proteins, as some are novel and allergenic responses are just starting to emerge. This does not mean new protein sources should not be explored, but that labeling should be clear so those who do develop an allergy know what is in them. 

## 6. Conclusions

Products made with plant-based protein and plant-based whole food diets are growing in popularity. Plant protein has been associated with benefits regarding health and physical function. The trend toward increasing plant protein intake is likely to continue as consumers expand their knowledge of the nutritional benefits of protein and sustainability concerns about the food supply are raised. Plant proteins may also become more valuable if current public health protein recommendations are revised upward. However, plant proteins differ in nutritional quality and those who choose to largely emphasize plant versus animal proteins need to be aware of these differences when planning an appropriate diet, especially in more vulnerable populations. In addition, potential safety issues have come to light and may continue to emerge with the increased amount, variety, and forms of plant proteins that are incorporated into the diet. More research is needed on the best ways to incorporate plant proteins into the diet safely and effectively.

## Figures and Tables

**Table 1:** Indispensable, dispensable, and conditionally indispensable amino acids in the human diet. Adapted from [11].

Indispensable	Dispensable	Conditionally Indispensable
Histidine	Alanine	Arginine
Isoleucine	Aspartic acid	Cysteine
Leucine	Asparagine	Glutamine
Lysine	Glutamic acid	Glycine
Methionine	Serine	Proline
Phenylalanine		Tyrosine
Threonine		
Tryptophan		
Valine		

**Table 2:** Protein quality of whey and selected vegetable protein sources.

Protein	PDCAAS ^1^	PDCAAS ^2^	PDCAAS ^3^	PDCAAS ^4^	DIAAS ^3^	Limiting Amino Acid(s), When Present	AA Profile: Materials Analyzed and References	Fractional Digestibility and References
Milk	1.00	1.00	1.00	1.00	1.08	None	Milk PC [20,21]	Fecal true protein: mean 0.96 [13,20] Ileal AA: range for individual AA 0.84–0.94 [22]
Whey	1.00	1.00	0.97	1.00	0.90	His	Whey PI [20,21,23] Whey PC [20,21]	Fecal true protein: mean 0.96 [20,24] Ileal AA: range for individual AA 0.89–1.00 [22]
Soy	0.99	1.00	0.93	1.00	0.92	SAA	Soy PI, Soy PC [25] Soy PI [20] Soy PI [21]	Fecal true protein: mean 0.97 [13,20,21,25] Ileal AA: range for individual AA 0.95–0.99 [22]
Canola	0.88	1.00	0.93	1.00	NA	AAA	Canola PI [26]; Canola PI [27]	Fecal true protein: mean 0.95 [26,27]
Potato	0.87	1.00	0.87	1.00	0.85	His	Solanic 100F Potato PI [28] Solanic 206P HMW and LMW [29] Potato protein [30] Potato juice protein concentrate [31] Avg of 6 potato varieties [32]	Fecal true protein: 0.89 [24] Ileal AA: range for individual AA 0.73–0.90 [22]
Pea	0.83	0.84	0.78	0.91	0.66	SAA *, Trp	Pea PC [33] Pea PC [21] Pea PC [20]	Fecal true protein: mean 0.97 [20,21,33] Ileal AA: range for individual AA 0.83–0.90 [20]
Quinoa	0.78	0.89	0.77	0.84	NA	Ile, Leu, Lys *, Thr, Val	Quinoa, raw [34] Quinoa [35] Quinoa from Salta [36] Uncooked quinoa [37] Field grown quinoa [38] Raw and unwashed quinoa [39]	Fecal true protein: mean 0.89 [39,40]
Chickpea	0.77	0.85	0.71	0.71	0.69	Leu, Lys, SAA *, Thr, Trp, Val	Boiled chickpeas [41]	Fecal true protein: 0.85 [42] Ilea AA: range for individual AA 0.72–0.9 [22]
Lentils	0.73	0.73	0.68	0.80	0.75	Leu, SAA *, Thr, Trp, Val	Lentils, mature seeds, ckd, bld without salt [37]	Fecal true protein: 0.85 [24] Ileal AA: range for individual AA 0.82–0.98 [22]
Red Kidney beans	0.68	0.68	0.63	0.74	0.61	Leu, Lys, SAA *, AAA, Thr, Trp, Val	Red kidney beans, cnd, drnd solids [37]	Fecal true protein: 0.81 [24] Ileal AA: range for individual AA 0.72–0.94 [22]
Fava/faba	0.63	0.65	0.60	0..67	NA	Lys, SAA *, Thr, Trp, Val	Fava bean PI [43] Cooked fava beans [44] Broadbeans, ckd [37] 8 faba cultivars [45] Faba bean PI [46]	Fecal true protein: 0.86 [47]
Barley	0.63	0.71	0.64	0.76	0.50	Lys *	Barley, pearled [48]	Fecal true protein: 0.98 [49] Ileal AA: range for individual AA 0.76–0.83 [22]
Pinto beans	0.61	0.61	0.57	0.66	NA	His, Ile, Leu, Lys, SAA *, AAA, Thr, Trp, Val	Pinto beans, cnd, drnd solids [37]	Fecal true protein: 0.73 [24]
Rice	0.53	0.60	0.54	0.64	0.52	Lys *, Thr	Rice PC [50] Rice endosperm protein [51] Oryzatein 90 and 80 Rice protein [52] Rice PC [21]	Fecal true protein: mean 0.90 [21,24,51] Ileal AA: mean ranges for individual AA 0.81–0.87 [21,22]
Oat	0.51	0.59	0.52	0.62	0.44	Lys *, Thr	Oat PC [53]; Rolled oats [21]	Fecal true protein: 0.91 [13] Ileal AA: range for individual AA 0.70–0.85 [22]
Peanut	0.46	0.52	0.47	0.55	0.47	Ile, Leu, Lys *, SAA, Thr, Trp, Val	Peanut PC and PI [54] Roasted peanuts [21]	Fecal true protein: 0.93 [24] Ileal AA: mean ranges for individual AA 0.82–0.96 [21,22]
Wheat	0.45	0.51	0.46	0.54	0.39	Ile, Leu *, Lys *, AAA, Thr *, Val	Whole meal and white flour [55] Wheat bran [21]	Fecal true protein: mean 0.94 [24] Ileal AA: mean ranges for individual AA 0.81–0.91 [22] (wheat bran, wheat flour, wheat gluten, wheat)
Corn	0.41	0.47	0.42	0.50	0.38	Ile, Lys *, SAA, Thr *, Trp*, Val	Corn meal [56] Corn tortillas [57]	Fecal true protein: 0.84 [24] Ileal AA: ranges for individual AA 0.75–0.88 [22]

^1^ FAO FN Paper 51 1989, ages 2–5 year, AA ref standard (mg/g protein) [13]: His 19, Ile 28, Leu 66, Lys 58, SAA 25, AAA 63, Thr 34, Trp 11, Val 35. ^2^ IOM 2002/2005, ages 1+ year, AA ref standard (mg/g protein) [11]: His 18, Ile 25, Leu 55, Lys 51, SAA 25, AAA 47, Thr 27, Trp 7, Val 32. ^3^ FAO FN Paper 92 2011, ages 0.5–3 year, AA ref standard (mg/g protein) [16]: His 20, Ile 32, Leu 66, Lys 57, SAA 27, AAA 52, Thr 31, Trp 8.5, Val 43. ^4^ FAO FN Paper 92 2011, older child, adolescent, adult, AA ref standard (mg/g protein) [16]: His 16, Ile 30, Leu 61, Lys 48, SAA 23, AAA 41, Thr 25, Trp 6.6, Val 40. PDCAAS, Protein Digestibility Corrected Amino Acid Score; DIAAS, Digestible Indispensable Amino Acid Score; AA, amino acid; His, histidine; Ile, isoleucine; Leu, leucine; Lys, lysine; SAA, sulfur amino acids (methionine and cysteine); AAA, aromatic amino acids (phenylalanine and tyrosine); Thr, threonine; TRP, tryptophan; Val, valine; PI, protein isolate; PC, protein concentrate; bld, boiled; ckd, cooked; cnd, canned; drnd, drained. * Limiting amino acid by all four amino acid reference standards.

**Table 3:** Glutamine concentration of selected plant and dairy proteins. Sources of data: References [68,69,70,71] and unpublished data.

Protein	Glutamine Concentration (mg/g Protein, Mean)	Glutamine Concentration (mg/g Protein, Range)
Wheat protein hydrolysate (*n* = 15)	296	184–402
Wheat protein isolate (*n* = 2)	208	184–232
Corn protein (*n* = 1)	196	--
Rice protein (*n* = 1)	130	--
Casein (*n* = 2)	102	100–104
Soy protein isolate (*n* = 2)	100	94–106
Soy protein concentrate (*n* = 1)	94	--
Milk protein concentrate (*n* = 1)	94	--
Whey protein concentrate (*n* = 2)	57	50–63
Ion exchange whey protein isolate (*n* = 1)	34	--
